# LSPR-Based Aptasensor for Rapid Urinary Detection of NT-proBNP

**DOI:** 10.3390/bios13070736

**Published:** 2023-07-17

**Authors:** Maria António, Rui Vitorino, Ana L. Daniel-da-Silva

**Affiliations:** 1 CICECO-Aveiro Institute of Materials, Department of Chemistry, University of Aveiro, 3810-193 Aveiro, Portugal; maantonio@ua.pt; 2 LAQV-REQUIMTE, Chemistry Department, University of Aveiro, 3810-193 Aveiro, Portugal; 3 iBiMED-Institute of Biomedicine, Department of Medical Sciences, University of Aveiro, 3810-193 Aveiro, Portugal; 4 UnIC@RISE, Department of Surgery and Physiology, Cardiovascular R&D Center, Faculty of Medicine, University of Porto, Alameda Professor Hernâni Monteiro, 4200-319 Porto, Portugal

**Keywords:** gold nanoparticles, aptamer, LSPR-based assay, NT-proBNP, urine

## Abstract

N-terminal pro-brain natriuretic peptide (NT-proBNP) is a myocardial stress biomarker that can be found in serum or plasma, saliva, and urine in the context of cardiovascular disease. In this study, we developed a rapid (~25 min) and straightforward localized surface plasmon resonance (LSPR)-based assay for detecting NT-proBNP in urine. The assay employs citrate-capped gold nanoparticles (AuNPs) and an aptamer specific for NT-proBNP, which initially interacts with NT-proBNP. The remaining unbound aptamer then interacts with the AuNPs, and the addition of NaCl induces the aggregation of the unprotected AuNPs, resulting in a decrease in absorbance at the LSPR band (A_521_) and an increase in absorbance at 750 nm (A_750_). The concentration of NT-proBNP showed a linear correlation with the aggregation ratio (A_521_/A_750_), and the assay demonstrated a limit of detection (LOD) of 0.303 µg·L^−1^ and a detection range of 0.566–8 µg·L^−1^. However, the presence of sulfur-containing proteins in saliva and fetal bovine serum hindered the detection of NT-proBNP in these biofluids. Nevertheless, the assay successfully detected NT-proBNP in diluted urine with an LOD of 0.417 µg·L^−1^ and a detection range of 0.589–6 µg·L^−1^. The observed values in urine samples from preterm infants with cardiovascular disease fell within this range, indicating the potential clinical relevance of the assay. The recovery percentages ranged from 92.3 to 116.3%. Overall, our findings suggest that the LSPR-based assay for NT-proBNP detection in urine can be a valuable tool for the diagnosis and treatment of cardiovascular disease.

## 1. Introduction

A biomarker is a measurable characteristic used to evaluate a normal or pathological process, which can be measured to identify the risk of disease development, disease diagnosis, or prognosis [1]. Several biomarkers associated with heart failure (HF) have been identified, such as cardiac troponin T, soluble ST2, interleukin-8, galectin-3, B-type natriuretic peptide (BNP), and NT-proBNP [2,3]. The NT-proBNP levels have been linked to acute congestive HF, with levels higher than 450 ng·L^−1^ for individuals under 50 years and over 900 ng·L^−1^ for those above 50 years old; levels lower than 300 ng·L^−1^ are considered normal [4]. NT-proBNP levels were found to be helpful for HF prognosis [5], and their decrease was associated with HF regression [6]. In addition, NT-proBNP levels are related to cardiovascular events (>2000–10,000 ng·L^−1^) and cardiovascular mortality (>2000–20,000 ng·L^−1^) [7]. This biomarker is also expressed in saliva and urine in the cardiovascular disease context. Salivary NT-proBNP has been recognized as a valuable biomarker for HF because due to its high levels in HF patients (18.3–748.7 ng·L^−1^) compared with the control group (<16 ng·L^−1^) [8,9]. High levels of urinary NT-proBNP have also been found in the Japanese population with cardiovascular events (≥43 ng·L^−1^) [10], in preterm infants suffering from patent ductus arteriosus (≥567 ng·L^−1^) [11], and with bronchopulmonary dysplasia associated to pulmonary hypertension (≥2345 ng·L^−1^) at 28 weeks gestational age [12]. The simplicity of collecting saliva and urine for biomarker analysis is well known [13], especially in preterm infants. 

Currently, NT-proBNP detection relies mainly on electrochemical assays that use different nanomaterials and antibodies as recognition elements [14,15,16]. However, these characteristics increase the complexity and cost of the detection approach. Aptamers have been explored as a lower-cost alternative for NT-proBNP detection [17,18]. However, the design of these approaches remains complex, and more straightforward strategies are desirable. Gold nanoparticles (Au NPs) are widely used in biosensing [19,20,21]. Gold colloids are morphologically and size controllable [22], biocompatible [23], easily surface modified [24], and their high surface-to-volume ratio enables an enriched surface modification [25]. Moreover, AuNPs exhibit unique optical properties, including the localized surface plasmon resonance (LSPR) band [26], which makes them attractive for biosensor development. While optical-based assays using AuNPs are commonly employed for biosensing purposes [27], there is currently no description of VIS-spectroscopy based assays for the detection of NT-proBNP. In the presence of the target, the interparticle distance decreases, resulting in the aggregation of modified gold nanoparticles [28,29]. Consequently, this aggregation induces a decrease in LSPR band intensity [30], which is often accompanied by a band-shift and the appearance of a new band at a higher wavelength [29].

In this study, we propose the development of a simple LSPR-based assay for quick urinary NT-proBNP detection using aptamer-modified gold nanoparticles and detection by VIS spectroscopy. With this approach, we intend to decrease the detection assay’s complexity, time-to-result, and cost.

## 2. Materials and Methods

### 2.1. Chemicals and Reagents

Tetrachloroauric (III) acid trihydrate (HAuCl_4_·3H_2_O, ≥99.9%), sodium citrate tribasic dihydrate (Na_3_C_6_H_5_O_7_·2H_2_O, ≥99.0%), phosphate-buffered saline (PBS) pH 7.4, trizma hydrochloride (C_4_H_11_NO_3_·HCl, 99%), bovine serum albumin (BSA) from bovine (≥98%), C-reactive protein (CRP) from human fluids (≥90%), urea (CH_4_ON_2_, 99%), α-amylase from porcine pancreas (≥10 units/mg) and fetal bovine serum were purchased from Sigma-Aldrich (St. Louis, MO, USA). Ethylenediaminetetraacetic acid (C_10_H_16_N_2_O_8_, 99%) was purchased from PanReac AppliChem. The aptamer specific for NT-proBNP was a single-stranded (ss) DNA with the following sequence (5′-GGCAGGAAGACAAACAGGTCGTAGTGAAACTGTCCACCGTAGACCGGTTATCTGTTGGTCTGTGGTGCTGT-3′, MW: 22,383.6 g·mol^−1^, ε: 705,500 L·mol^−1^·cm^−1^) purchased from Eurogentec. The NT-proBNP (HPLGSPGSASDLETSGLQEQRNHLQGKLSELQVEQTSLEPLQESPRPTGVWKSREVATEGIRGHRKMVLYTLRAPRSPKMVQGSGCFGRKMDRISSSSGLGCKVLRRH, >95%) was acquired from abcam. The galectin-3 (98%) was purchased from RayBiotech. The reagents were used as acquired. Ultra-pure water was obtained using the Milli-Q system using a 0.22 µm filter (Synergy equipment, Millipore (Burlington, MA, USA)).

### 2.2. Synthesis of Gold Nanoparticles

Gold nanoparticles were synthesized using the Turkevich method, following the procedure described by Grabar et al. [31]. Briefly, a HAuCl_4_ solution (100 mL, 1 mM) was brought to boil under vigorous stirring and reflux. Subsequentially, a sodium citrate solution (10 mL, 38.8 mM) was added to the gold solution, and the mixture was allowed to react for 10 min until the color changed from pale yellow to reddish-purple. The heat was then turned off, and the colloid was left to cold until room temperature under continuous stirring. Subsequently, the AuNPs were stored in an opaque plastic vial at 4 °C until they were used. The AuNPs were used as synthesized samples.

### 2.3. Preparation of Aptamer Solution and NT-proBNP Solution Series

The NT-proBNP was centrifuged at 16,300× *g* for 2 min to form a pellet at the bottom of the vial. A certain volume of 5 mM PBS pH = 7.4 was added to the NT-proBNP to prepare a stock solution of 10 mg·L^−1^. The resulting NT-proBNP solution was divided into 25 µL fractions and stored at −80 °C until needed. Prior to use, the NT-proBNP solution was thawed on ice and diluted in 5 mM PBS pH = 7.4 to the desired concentration of 0.5, 1, 2, 4, 6, 8, 10, 12, 14, and 16 µg·L^−1^. 

The NT-proBNP aptamer was prepared by dissolving it in a solution of 10 mM Tris-HCl with 0.2 mM of EDTA to a concentration of 100 µM, following the manufacturer’s instructions. The aptamer solution was stored at −20 °C until use, and further dilutions were prepared in Tris-HCl with EDTA.

### 2.4. Procedure for Detecting NT-proBNP Using AuNPs

In a typical experiment, 50 µL of NT-proBNP (ranging from 0 to 16 µg·L^−1^) was combined with 50 µL of 0.25 µM aptamer and allowed to react for 15 min at 25 °C. Subsequently, 50 µL of AuNPs was added and incubated for 5 min. Next, 40 µL of 0.2 M NaCl was added to the colloid and allowed to react for 4 min. A volume of 150 µL of the resulting solution was transferred to a microplate and analyzed by UV-VIS spectrophotometry to determine the aggregation ratio, A_750_/A_521_. The absorbance values at 750 nm and 521 nm were denoted as A_750_ and A_521_, respectively.

### 2.5. Characterization of the Aptamer and NT-proBNP by Circular Dichroism

Solutions of aptamer (25 µM) and NT-proBNP (9 µg·L^−1^) were prepared and analyzed using the circular dichroism (CD) technique. The Apt-NT-proBNP was prepared by adding 30 µL of aptamer (25 µM) to NT-proBNP (9 µg·L^−1^) and allowing it to react for 25 min at 325 rpm at 25 °C. Then, 300 µL of Milli Q water was added, and the resulting solution was transferred to a cell for CD analysis.

### 2.6. Specificity for NT-proBNP

BSA, α-amylase, urea, Gal-3, and CRP were used to evaluate the specificity of the developed method for NT-proBNP. Each potential interferent was tested at a concentration of 2 mg·L^−1^ in the presence of NT-proBNP (2 µg·L^−1^). The assay involved adding 50 µL of a mixture of interferent and NT-proBNP to 50 µL of 0.25 µM aptamer, following the protocol as described above.

### 2.7. Collection of Urine and Saliva

To assess the potential matrix interference of saliva and urine in the described method, samples of both were collected and pre-treated. Saliva samples were collected 1 h after breakfast and tooth brushing in an Eppendorf tube (about 1 mL of sample). The collected saliva was immediately placed in an ice bath until centrifugation, which was performed at 4 °C for 20 min at 12,000× *g*. After centrifugation, the clear supernatant was equally distributed into Eppendorf tubes and stored at −20 °C until further use. For urine samples, the first-morning urine was collected in a falcon tube (about 15 mL of the sample) and kept in an ice bath until centrifugation. The sample was then centrifuged at 4 °C for 10 min at 3000× *g*, and the recovered supernatant was placed into Eppendorf tubes, equally distributed, and frozen at −20 °C until use.

### 2.8. Calibration Curves for NT-proBNP in Biofluids

NT-proBNP was detected in various biofluids, including urine, saliva, and FBS, as well as the PBS buffer. To prepare samples for analysis, different concentrations of NT-proBNP (0–8 µg·L^−1^) were added to diluted urine (1:100), saliva (1:10), and fetal serum bovine (1:100). Prior to analysis, the biofluids were defrosted in an ice bath and diluted using Milli-Q water.

### 2.9. Instrumentation

The AuNPs were analyzed by UV-VIS spectroscopy using a Multiskan GO microplate spectrophotometer at 25 °C. The UV-VIS spectra were obtained in fast mode and with a bandwidth of 1 nm, using UV-Star 96-well microplates from Greiner. 

To determine the surface charge and hydrodynamic diameter (HD) of the AuNPs, electrophoretic light scattering and dynamic light scattering (DLS) were conducted using a Zetasizer Nano ZS instrument from Malvern Panalytical. The instrument was equipped with a HeNe laser (633 nm) and a scattering detector (173°) for zeta potential measurements. DLS, pH measurement, and zeta potential (ZP) analysis were performed on as-synthesized AuNPs.

A Hitachi HD-2700 scanning transmission electron (STEM) microscope operated at 200 kV was utilized to assess the size and morphology of the AuNPs. Samples for STEM analysis were prepared by evaporating a drop of the colloid on carbon-coated copper grids. The particle size histogram was built by analyzing STEM images with the ImageJ software (version 1.46).

The concentration of Au in the Au colloids was determined by inductively coupled plasma mass spectrometry (ICP-MS). To digest the sample, 10 μL of HNO_3_ (65%) and 30 μL of HCl (37%) were added to 0.25 mL of AuNPs at RT for more than 48 h.

The reaction between AuNPs, aptamer and NT-proBNP was conducted in the orbital shaker incubator (IKA KS 4000 i) at 27 °C and 300 rpm.

Circular dichroism (CD) measurements were conducted to study the G-quadruplex structure of the aptamer, NT-proBNP, and the aptamer in the presence of NT-proBNP. CD spectra were obtained using quartz cell in a Jasco J-1500 CD spectrometer at 50 nm/min, with 4 s of digital integration time, 1 nm of bandwidth, and 4 scans, as suggested by Jasco for biological samples analysis. The wavelength used ranged from 190 to 300 nm.

### 2.10. Statistical Analysis

The limit of detection (LOD) and limit of quantification (LOQ) were determined from the linear correlation between A_750_/A_521_ values and NT-proBNP concentration [32,33] using Equations (1)–(5).

(1) $$y_{B}=\sum y_{i}-(r\cdot \sum x_{i})$$

(2) $$S_{y/x}=\sqrt{\frac{{\left(y_{i}-y_{calc}\right)}^{2}}{\left(N-2\right)}}$$

(3) $$y_{calc}=\left(r\cdot x_{i}\right)+y_{B}$$

(4) $$LOD=3(S_{y/x}/r)$$

(5) $$LOQ=10(S_{y/x}/r)$$

The *x_i_*, *y_i_*, *r*, and *N* are the concentration of NT-proBNP, values of A_750_/A_521_, slope, and the number of points of the calibration curve, respectively. 

## 3. Results and Discussion

### 3.1. Characterization of Gold Nanoparticles

**Figure 1:**
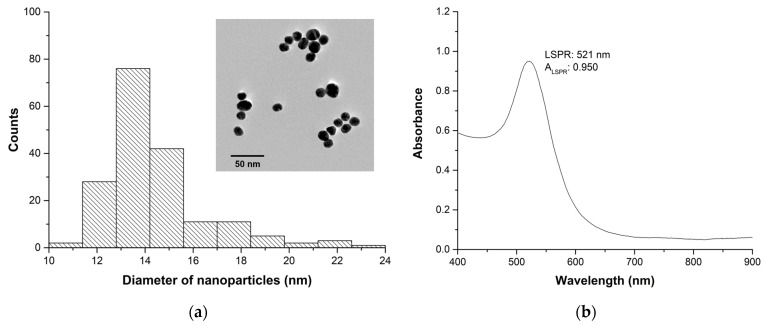
(**a**) Histogram and STEM image (inset) and; (**b**) UV-VIS spectrum of AuNPs.

In this study, citrate-capped AuNPs were synthesized using the Turkevich method, resulting in spheroidal particles with an average size of 14.4 ± 2.1 nm, as determined by STEM analysis. The histogram of the diameter of AuNPs and a representative STEM image are shown in Figure 1a. The UV-VIS spectroscopy analysis of the AuNPs (diluted 1:3) demonstrated the presence of the LSPR band at 521 nm with an absorbance of 0.950, as illustrated in Figure 1b.

The concentration of AuNPs in the colloid was determined to be 0.104 nM, with a total Au concentration of 0.960 mM, as measured by ICP-MS analysis. The NPs concentration was calculated considering the spheroidal morphology and cubic crystalline structure of Au. The surface charge of the AuNPs was determined to be −66.4 ± 1.7 mV at pH 5.7 using ZP measurements, which is attributed to the capping agent, citrate. The negative charge of citrate on the surface of the AuNPs provides colloidal stability by preventing aggregation due to electrostatic repulsion. The HD of the AuNPs was found to be 21.6 ± 0.5 nm, which is larger than the average size due to the presence of citrate molecules on the surface. The polydispersity index (PDI) of the AuNPs was 0.272, suggesting a moderately polydisperse colloid [34]. Table S1 of the Supplementary Material summarizes the most important characteristics of AuNPs.

### 3.2. Detection of NT-proBNP Using Aptamer and AuNPs

**Figure 2:**
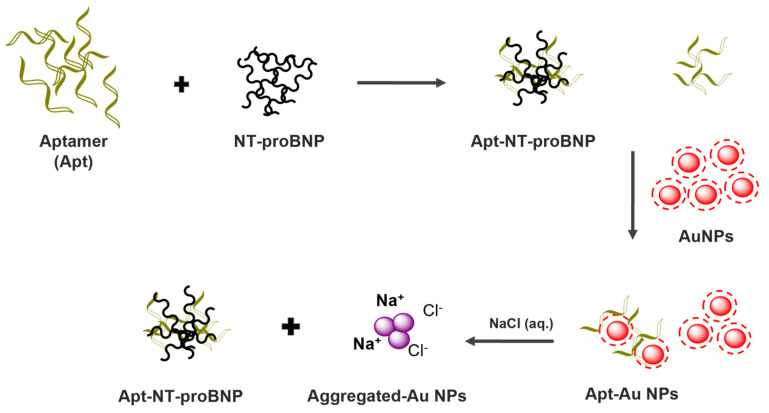
Schematic illustration of the principle of NT-proBNP detection.

Figure 2 depicts the working principle of the developed strategy. Initially, the aptamer and NT-proBNP interact with each other. Subsequently, citrate-capped AuNPs were added. Any excess of aptamer molecules that did not react with NT-proBNP may interact with the AuNPs, preventing their aggregation in the presence of NaCl. As the concentration of NT-proBNP increases, more of the available aptamer molecules bind to NT-proBNP, reducing the amount of free aptamer molecules that can interact with the AuNPs. This makes the AuNPs more likely to aggregate in response to NaCl, providing a detectable signal that can be correlated to the concentration of NT-proBNP.

Based on our previous findings [29], it is suggested that the aptamer could potentially engage in various interactions such as van der Waals forces, hydrophobic interactions, and electrostatic interactions with citrate-capped AuNPs. In this context, the lengthy structure of the aptamer is anticipated to impede its detachment from the AuNPs, even when NaCl is present.

To establish favorable conditions to perform detection, we have screened the effect of the aptamer concentration, the time of incubation of the aptamer with NT-proBNP, and the amount of NaCl on the optical response. The effect of these parameters in the A_750_/A_521_ ratio was evaluated in the absence (0 µg·L^−1^) and presence of NT-proBNP (0.5 µg·L^−1^). Our results (Figure S1a, Supplementary Material) show that a concentration of 0.25 µM of the aptamer provided a greater difference between the A_750_/A_521_ values at NT-proBNP concentrations of 0 and 0.5 µg·L^−1^. An incubation time of 15 min was set, because longer times resulted in a decrease in the difference between aggregation ratios (Figure S1b, Supplementary Material). The volume of 40 µL of 0.2 M of NaCl was chosen, because higher volumes resulted in similar differences in the A_750_/A_521_ ratio (Figure S1c, Supplementary Material). We note that the increase in the aptamer concentration led to a decrease in the A_750_/A_521_ values (less aggregation), namely in the absence of NT-proBNP (0 µg·L^−1^) (Figure S1a, Supplementary Material). This observation provides evidence that the aptamer plays a role in preventing AuNPs aggregation triggered by NaCl. 

**Figure 3:**
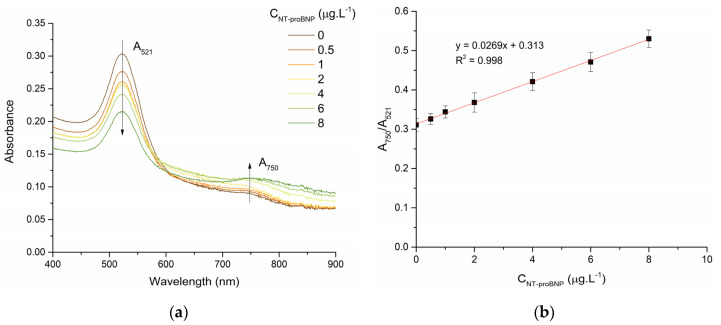
(**a**) VIS spectra of Apt-AuNPs in the presence of different concentrations of NT-proBNP and; (**b**) Calibration curve of NT-proBNP (0-8 µg.L^−1^) in PBS buffer.

The working principle of the assay was demonstrated by measuring the absorbance spectra of Apt-AuNPs after the addition of different concentrations of NT-proBNP, as shown in Figure 3a. With increasing concentration of NT-proBNP, we observed a decrease in the absorbance of the LSPR band (521 nm) and an increase in absorbance at 750 nm, indicating the salt-induced aggregation of AuNPs. The A_750_/A_521_ was plotted against different concentrations of NT-proBNP. A linear relationship was obtained between A_750_/A_521_ and the NT-proBNP concentrations from 0.5 to 8 µg·L^−1^ with a correlation coefficient (R^2^) of 0.998 (Figure 3b). The LOD and LOQ were found to be 0.303 and 0.566 µg·L^−1^, respectively. The coefficient of variation of the developed method was found to range from 4.23 to 6.63%.

### 3.3. Interaction of Aptamer with NT-proBNP

The dissociation constant (k_d_) for the interaction between the aptamer and NT-proBNP was determined to be 1.38 × 10^−12^ M. This value was obtained by plotting the A_750_/A_521_ ratios against NT-proBNP concentrations ranging from 0 to 0.6 nM (8 µg·L^−1^) (Figure S2, Supplementary Material) and fitting the data with the Hill model (Equation (S1), Supplementary Material). The Hill coefficient (n) was found to be 0.182, indicating negative cooperativity binding in the binding of NT-proBNP to the aptamer.

**Figure 4:**
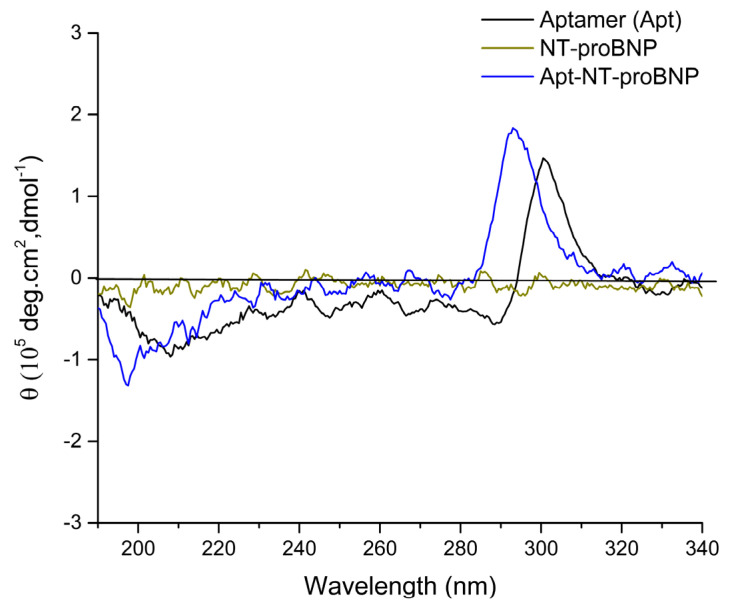
Circular dichroism spectra of aptamer, NT-proBNP and Apt-NT-proBNP.

Circular dichroism measurements were performed to investigate the formation of the Apt-NT-proBNP complex. The CD spectra of Apt, NT-proBNP, and Apt-NT-proBNP are shown in Figure 4. Given that the aptamer is a single-stranded DNA sequence enriched in guanine bases, we expect it to form a G-quadruplex (G4) structure. The CD spectrum of the aptamer revealed a minor negative band spanning 190 to 290 nm and a significant positive band at about 300 nm, indicating the formation of an antiparallel G4 structure [35]. In contrast, the NT-proBNP did not exhibit any CD signal, which was likely due to its peptide nature lacking a defined secondary structure. However, the CD analysis of Apt-NT-proBNP showed a blue shift in both the negative and positive bands and an increase in ellipticity (θ) values, suggesting changes in the secondary structure of the aptamer G4 [36,37]. These changes may be due to the formation of an Apt-NT-proBNP complex. Moreover, our previous investigation demonstrated that the presence of NaCl in the Apt-NT-proBNP solution did not affect the G4 structure [29].

### 3.4. Selectivity Studies

**Figure 5:**
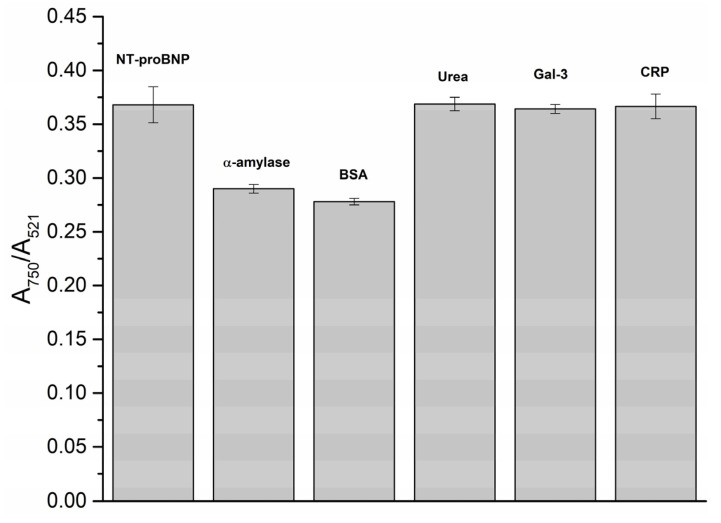
Aggregation ratio of Apt-AuNPs in the presence of distinct biomolecules and NT-proBNP.

To evaluate the specificity of the method toward NT-proBNP, BSA, α-amylase, Gal-3, CRP, and urea were employed in this study. Figure 5 shows the A_750_/A_521_ of Apt-AuNPs after incubating with each biomolecule in the presence of NT-proBNP. The interference from urea, Gal-3, and CRP in the A_750_/A_521_ was smaller compared to that from α-amylase and BSA. The presence of α-amylase and BSA resulted in A_750_/A_521_ values (0.290, 0.278, respectively) lower than the A_750_/A_521_ value observed for 0 µg.L^−1^ NT-proBNP (0.311) on the calibration curve (Figure 3b), indicating the interference of both proteins in the method. However, for urea, Gal-3, and NT-proBNP, the coefficient of variation (CV) and recovery (%) were calculated and found to be in the ranges of 8.5–8.8%, 6.1–8.3%, and 9.5–10.5%, and 90.1–112.7%, 85.4–100.8%, and 86.3–118.1%, respectively. These results indicate a similar analytical performance of Apt-AuNPs in the detection of NT-proBNP in the presence of urea, Gal-3, and CRP.

### 3.5. Effect of Biofluids on NT-proBNP Detection

To evaluate the effectiveness of the developed method in analyzing complex matrices, NT-proBNP was spiked into diluted urine, saliva, and FBS. Figure S3 of the Supplementary Material displays the measured A_750_/A_521_. In diluted urine, the A_750_/A_521_ values were higher compared to those observed in buffered NT-proBNP (Figure 3b). For instance, in the absence of NT-proBNP, the A_750_/A_521_ was 0.311 ± 0.0168 in buffer and 0.481 ± 0.006 in diluted urine, suggesting that the ionic species in urine might contribute to higher A_750_/A_521_ values. Additionally, using the Hill model (Figure S4, Supplementary Material), we obtained a k_d_ value of 2.71 × 10^−9^ M and a Hill coefficient (n) of 0.0751 in diluted urine. Notably, a negative cooperativity binding of the aptamer with the NT-proBNP was observed, which was similar to what was observed in buffered NT-proBNP. The A_750_/A_521_ increased progressively with the NT-proBNP concentration up to 6 µg·L^−1^. Conversely, in diluted saliva and FBS, the A_750_/A_521_ values were lower than those in buffered NT-proBNP, and increasing NT-proBNP concentrations did not produce significant changes in the AR (Figure S3, Supplementary Material). These findings are consistent with the interference observed with BSA and α-amylase, since FBS contains albumin and saliva has albumin and α-amylase. The colloidal stabilization of AuNPs can be explained by the interaction between sulfur-containing amino acids in proteins like albumin and α-amylase with AuNPs via Au-S bonds [38,39,40].

### 3.6. Detection of NT-proBNP in Urine

**Figure 6:**
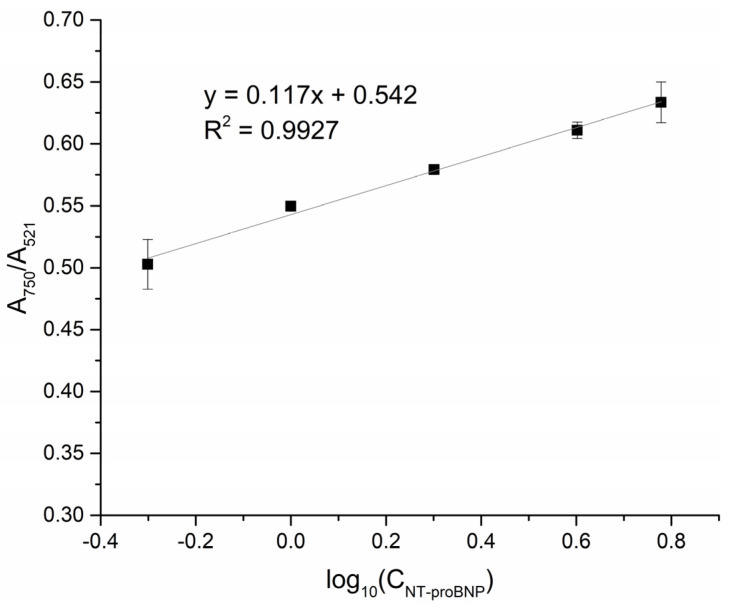
Calibration curve of NT-proBNP (0.5–6 µg·L^−1^) in urine.

As mentioned above, NT-proBNP could be detected in urine. Figure 6 shows a linear correlation between the A_750_/A_521_ values and the logarithmic NT-proBNP concentration for concentrations ranging from 0 to 6 µg·L^−1^. The LOD was determined to be 0.417 µg·L^−1^ and the LOQ was 0.589 µg·L^−1^. The CV ranged from 0.47 to 4.02% and the recovery percentage ranged from 92.3 to 116.3%.

Commercially available ELISA kits for detecting NT-proBNP show an LOD and LOQ range of 0.0115–0.14 µg·L^−1^ [41,42] and 0.0219–0.312 µg·L^−1^ [41,43], respectively. The LOD and LOQ values obtained from our method are within the same order of magnitude as some ELISA kits (Table 1). Furthermore, our method produced results for 96 tests about 3 to 18 times faster and at a cost that is 4 to 35 times less expensive than the available commercial kits.

## 4. Conclusions

Our study presents a successful and innovative approach for the rapid detection of NT-proBNP in urine using citrate-capped AuNPs and an NT-proBNP-specific aptamer. The high binding affinity between the aptamer and NT-proBNP, with a dissociation constant of 1.38 × 10^−12^ M, allowed the detection of NT-proBNP at concentrations up to 8 µg·L^−1^ in buffered NT-proBNP. The limit of detection (LOD) and limit of quantification (LOQ) for the assay were 0.303 µg·L^−1^ and 0.566 µg·L^−1^, respectively. Our results indicate that the assay is suitable for use with urine samples because other biofluids such as saliva and FBS lead to the colloidal stabilization of AuNPs, which hinders the detection of NT-proBNP. We were able to detect NT-proBNP in diluted urine samples at concentrations up to 6 µg·L^−1^ with an LOD and LOQ of 0.417 µg·L^−1^ and 0.589 µg·L^−1^, respectively. These results suggest that our aptamer-based LSPR assay is a promising tool for the diagnosis and monitoring of cardiovascular disease through the detection of NT-proBNP in urine samples. This assay has the potential to be particularly useful in preterm infants with cardiovascular disease in whom urine levels of NT-proBNP fall within the range of this assay. Overall, our study represents a valuable contribution to the diagnosis of cardiovascular disease.

## Figures and Tables

**Table 1:** Comparison of the method reported in this work with ELISA commercial kits.

Method	LOD	Detection Range	Time to Result	Cost (96 Tests)	Ref.
ELISA (1)	11.5 ng·L^−1^	21.9–1400 ng·L^−1^	1 h 30	740 euros	[41]
ELISA (2)	0.14 µg·L^−1^	0.137–100 µg·L^−1^	4 h 45	479 euros	[42]
ELISA (3)	-	0.312–10 µg·L^−1^	~9 h	76 euros	[43]
Apt-AuNPs	0.417 µg·L^−1^	0.589–6 µg·L^−1^	~25 min	~15 euros ^1^	This work

^1^ estimated costs including AuNPs, aptamer, and other reactants.

## Data Availability

The data presented in this study are available in the present article and in the supplementary material.

## Supplementary Materials

The following supporting information can be downloaded at: https://www.mdpi.com/article/10.3390/bios13070736/s1, Table S1: pH, Zeta potential, hydrodynamic diameter (HD) and PDI of AuNPs as synthesized; Figure S1: Effect of (a) concentration of aptamer, (b) time of incubation and (c) volume of NaCl in the A_750_/A_521_ ratio in NT-proBNP detection; Figure S2: Fitting curve (solid line) of Hill equation for determining k_d_; Figure S3: Plot of the aggregation ratio versus NT-proBNP concentration in spiked urine saliva and FBS; Figure S4: Curve fitting (solid line) of the Hill equation to determine the dissociation constant (k_d_) for the interaction between Apt and NT-proBNP in urine.
