# Supplementary material for: LSPR-Based Aptasensor for Rapid Urinary Detection of NT-proBNP
Source: Biosensors (Basel). 2023 Jul 17;13(7):736. doi: 10.3390/bios13070736 (PMC10377000; doi:10.3390/bios13070736)
Supplement: Supplementary file 1 [file biosensors-13-00736-s001.zip › biosensors-2458481-supplementary.pdf]

Supplementary Material

LSPR-based Aptasensor for Rapid Urinary Detection of NT-proBNP

Maria António[1], Rui Vitorino[3,4,5,*] and Ana L. Daniel-da-Silva[1,*]

[1]CICECO-Aveiro Institute of Materials, Department of Chemistry, University of Aveiro, 3810-193 Aveiro, Portugal

[2]LAQV-REQUIMTE, Chemistry Department, University of Aveiro, 3810-193, Aveiro, Portugal

[3]iBiMED-Institute of Biomedicine, Department of Medical Sciences, University of Aveiro, Aveiro, 3810-193, Portugal

[4]UnIC@RISE, Department of Surgery and Physiology, Cardiovascular R&D Center, Faculty of Medicine of the University of Porto, Alameda Professor Hernâni Monteiro, 4200-319 Porto, Portugal

CONTENTS:

S1. Characterization of AuNPs……………………..……………..…………… S3

S2. Detection of NT-proBNP using aptamer and AuNPs......................... S4

S3. Interaction of aptamer with NT-proBNP….……………………..………. S5

S4. Effect of biofluids on NT-proBNP detection……..………………...….. S6

S.1. Characterization of AuNPs

Table S1: pH, Zeta potential, hydrodynamic diameter (HD) and PDI of AuNPs as synthesized.

pH	Zeta potential (mV)	HD (nm)	PDI
5.7	- 66.4±1.7	21.6 ± 0.5	0.272

S.2. Detection of NT-proBNP using aptamer and AuNPs

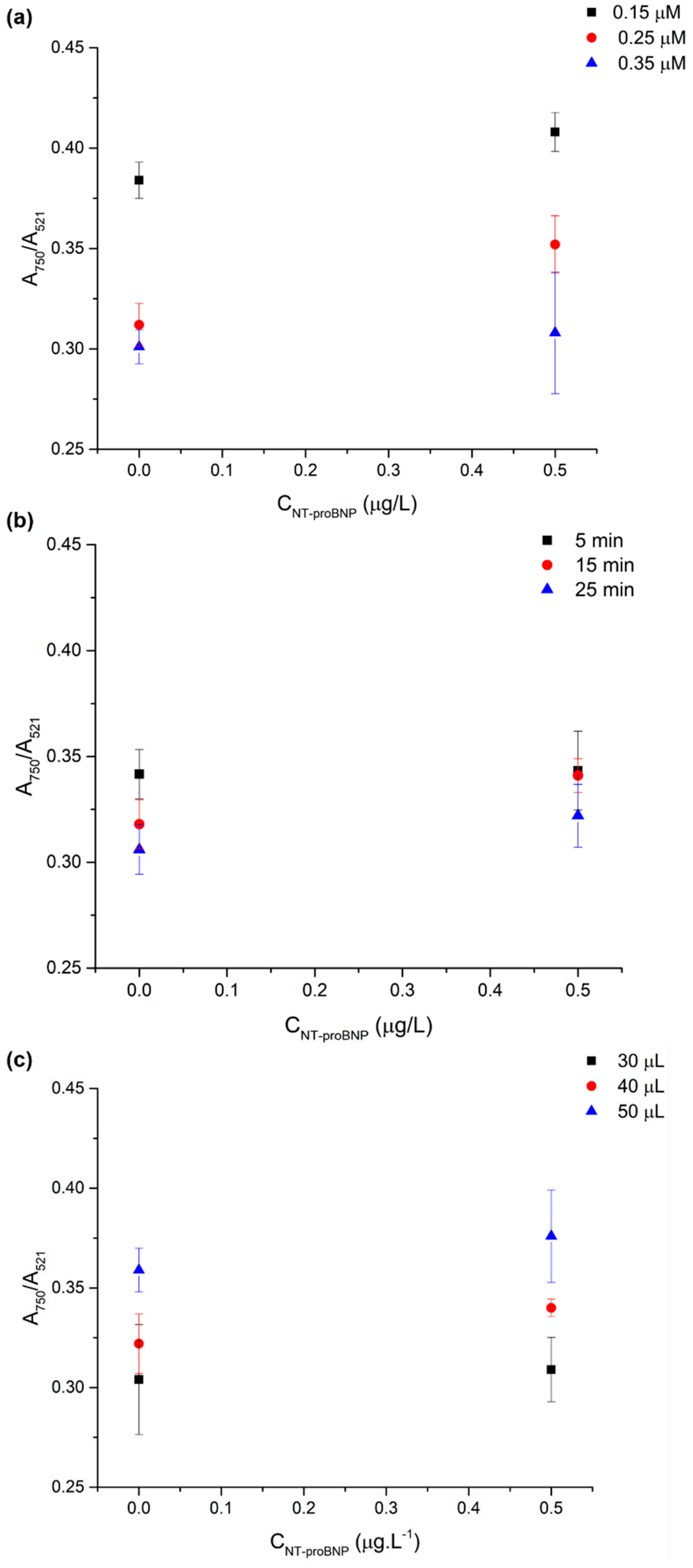

Figure S1: Effect of **(a)** concentration of aptamer, **(b)** time of incubation and **(c)** volume of NaCl in the A750/A521 ratio in NT-proBNP detection.

S.3. Interaction of aptamer with NT-proBNP

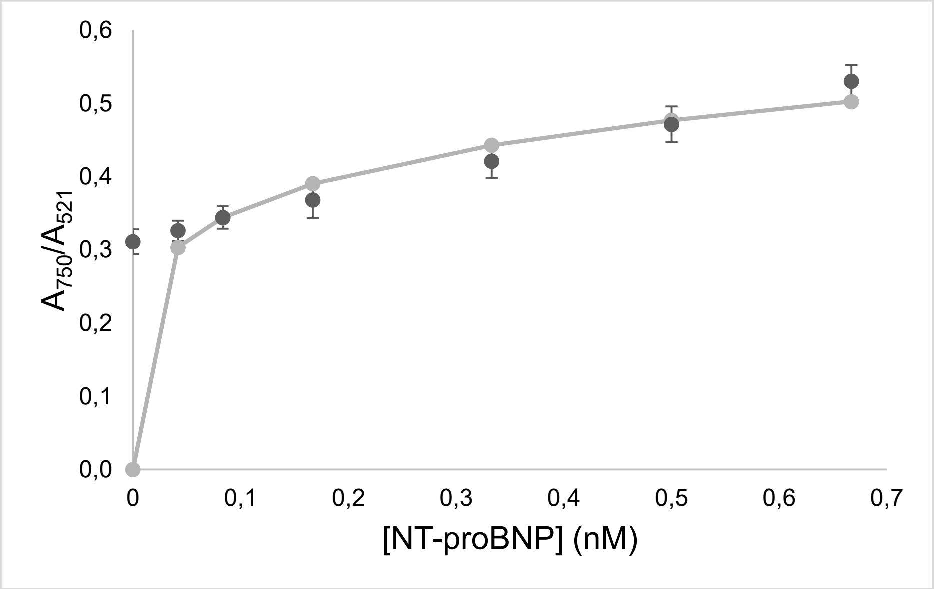

Figure S2: Fitting curve (solid line) of Hill equation for determining k_d.

$$Y = \frac{B_{max} \cdot X^n}{k_d^n + X^n} \quad \text{(S1)}$$

Equation S1 is the Hill equation where Y presents for the A_{750}/A_{521}, X is the initial concentration of NT-proBNP in nM, B_{max} is the maximum specific binding in nm, n is the Hill coefficient and k_d is the apparent dissociation constant.

S4. Effect of biofluids on NT-proBNP detection

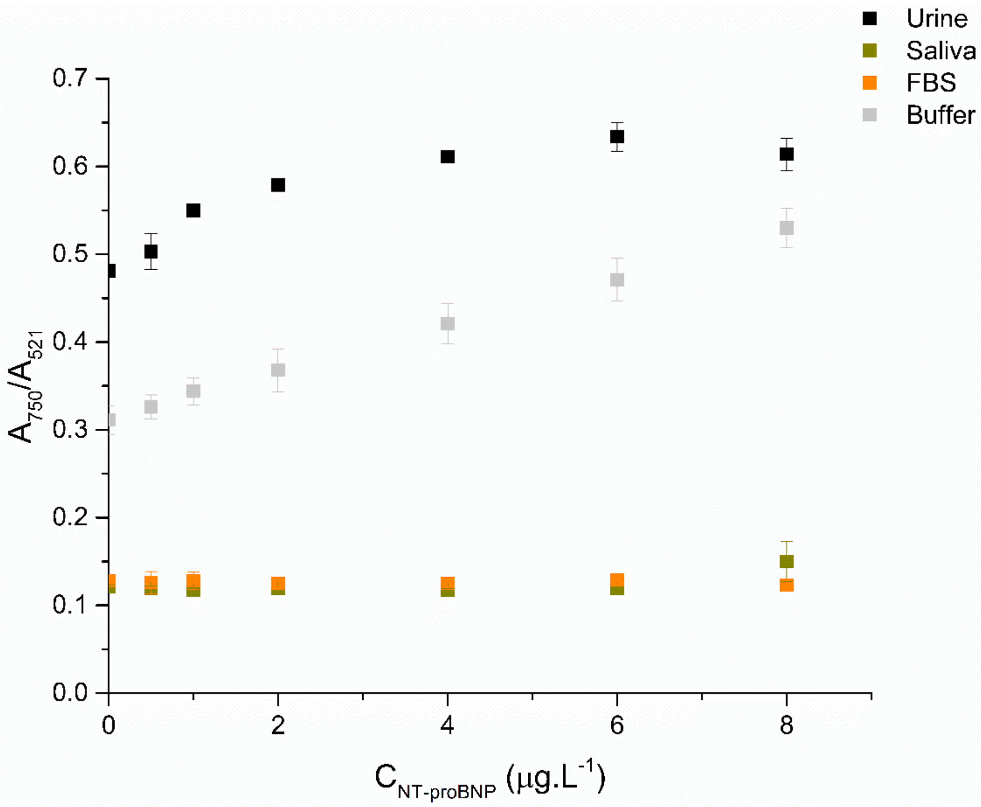

Figure S3: Plot of the aggregation ratio versus NT-proBNP concentration in spiked urine saliva and FBS.

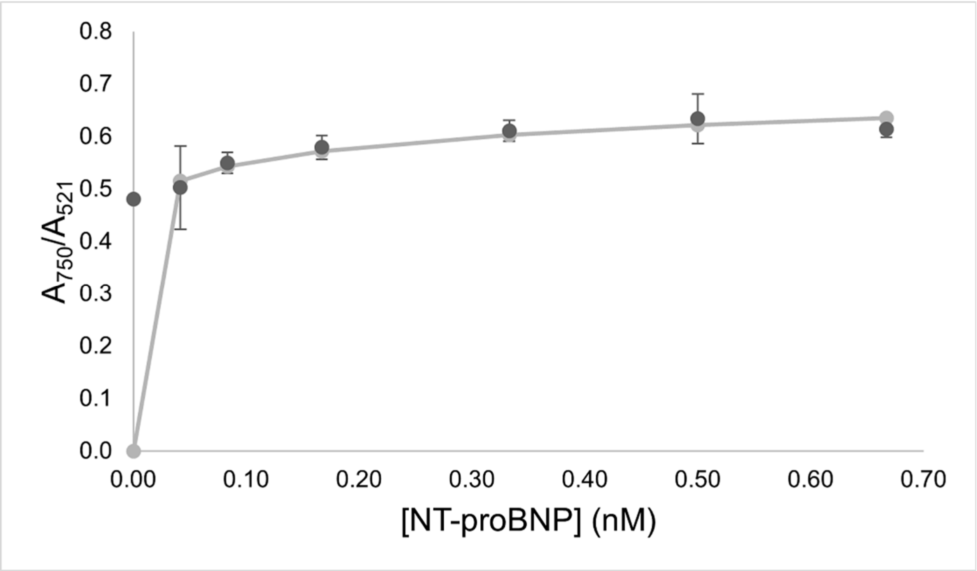

Figure S4: Curve fitting (solid line) of the Hill equation to determine the dissociation constant (k_d) for the interaction between Apt and NT-proBNP in urine.
